# Sleep abnormalities associated with alcohol, cannabis, cocaine, and opiate use: a comprehensive review

**DOI:** 10.1186/s13722-016-0056-7

**Published:** 2016-04-26

**Authors:** Gustavo A. Angarita, Nazli Emadi, Sarah Hodges, Peter T. Morgan

**Affiliations:** Yale University Department of Psychiatry, Connecticut Mental Health Center, 34 Park Street, New Haven, CT 06519 USA

**Keywords:** Sleep, Insomnia, Alcohol, Cannabis, Cocaine, Opiates

## Abstract

Sleep abnormalities are associated with acute and chronic use of addictive substances. Although sleep complaints associated with use and abstinence from addictive substances are widely recognized, familiarity with the underlying sleep abnormalities is often lacking, despite evidence that these sleep abnormalities may be recalcitrant and impede good outcomes. Substantial research has now characterized the abnormalities associated with acute and chronic use of alcohol, cannabis, cocaine, and opiates. This review summarizes this research and discusses the clinical implications of sleep abnormalities in the treatment of substance use disorders.

## Background

Sleep problems are commonly associated with drug and alcohol use. Nearly 70 % of patients admitted for detoxification report sleep problems prior to admission, and 80 % of those who report sleep problems relate them to their substance use [169]. The association between substance use and sleep problems appears to be bidirectional [105, 110], with sleep problems increasing risk for developing substance use disorders [31, 89, 210], and acute and chronic substance use leading to acute and chronic problems with sleep [44, 47, 89, 97, 104, 138, 156, 168]. Evidence also indicates that long-term abstinence from chronic substance use can reverse some sleep problems [13, 37]. This paper aims to explore and clarify the strong yet not entirely understood connection between abnormalities in sleep and substance use. By improving our understanding of sleep disorders that either predispose to substance use or are the result of chronic substance use, we may be better able to prevent and treat substance use disorders.

Understanding the sleep problems related to substance use disorders requires characterizing them both subjectively and objectively, while considering how sleep responds to periods of use and abstinence. This review will describe such research with regard to alcohol, cannabis, cocaine, and opioids. In addition, this review will discuss evidence that sleep abnormalities predict use and relapse, and that sleep abnormalities can be modulated to improve clinical outcome. This paper will also review potential pharmacological agents that modulate sleep. Psychotherapy options, albeit evidence-based and of clear clinical value, will not be discussed in this review as these are addressed elsewhere [15, 110].

## Methods

This is a narrative, non-systematic review of clinical trials conducted in humans. For the literature search, Pubmed, Ovid Medline, and Web of Science databases were used. For each drug (e.g., alcohol, cannabis/marijuana, cocaine, and opioids/heroin) keywords included terms describing abnormal/pathological use (e.g., alcohol use disorders, alcohol abuse, alcohol dependence, and alcohol addiction, etc.) combined with terms referring to sleep or sleep abnormalities [e.g., sleep, insomnia, polysomnography, total sleep time, slow-wave sleep, rapid eye movement (REM) sleep, sleep latency, REM latency; these terms are defined in Table 1]. In addition to extracting data available in each of the retrieved articles, reference lists from each retrieved article were examined to identify articles missed by the initial search. For each drug, the available literature on subjective measurements, objective measurements, the relationship between subjective and objective measurements, clinical and laboratory correlates of sleep outcomes, and pharmacotherapies related to sleep were summarized.

**Table 1:** Sleep terminology

Homeostatic sleep drive	The drive to sleep that progressively builds with continued wakefulness
Insomnia	A sleep disorder in which the quantity or quality of sleep is less than desired, usually characterized by difficulty falling or staying asleep, or waking too early, and experiencing daytime consequences of reduced sleep
Polysomnography (PSG)	A technique that records brain activity, eye movements, and muscle tone in order to study sleep and diagnose sleep disorders
Rapid eye movement (REM) sleep	The phase of sleep characterized by conjugate eye movements, paralysis of other muscles, and brain activity that is most similar to wakefulness
REM density	The frequency of rapid eye movements occurring during REM sleep. REM density increases over the course of the sleep period and is greatest when homeostatic sleep drive (sleep pressure) is lowest
REM latency	The amount of time from the onset of sleep to the onset of REM sleep
REM rebound	The characteristic increase in REM sleep after REM sleep deprivation
Self-administration	A method involving research participants administering a substance to themselves under observation in a clinical setting
Sleep architecture	The structure of sleep, including non-REM (stages N1, N2, and N3) and REM (stage R) sleep
Sleep efficiency (SE)	The percent of time in bed spent sleeping, calculated as total sleep time divided by time in bed
Sleep fragmentation	Disruption in sleep characterized by awakenings and transitions to light (stage N1) sleep from deeper sleep
Sleep latency (SL)	The amount of time from lights out to sleep onset
Slow-wave sleep (SWS)	Also known as stage N3 sleep, slow wave sleep is characterized by low frequency and high amplitude waves
Total sleep time (TST)	The amount of sleep in one complete episode of sleeping, usually reported in minutes
Wake after sleep onset (WASO)	The amount of time awake after the onset of sleep and before final wakening

## Alcohol

### Subjective measurements

Alcohol is widely used as a sleep-promoting agent. However, as the consumption of alcohol becomes chronic, alcohol has less of an hypnotic effect [196]. Significant, self-reported sleep problems are highly prevalent among alcohol users with rates of clinical insomnia between approximately 35 and 70 % depending on the setting and stage of use, among other parameters [35, 48]. These rates are substantially higher than those observed in the general population (i.e. ~15 to 30 %) [32]. Complaints typically include difficulty falling asleep, frequent awakenings, daytime sleepiness, and abnormal sleep quality [15, 34, 196], but could also include hypersomnia [196]. Notably, sleep complaints associated with alcohol use disorders are one of the most refractory problems to resolve [34, 69, 82], and insomnia is the most frequent complaint among alcoholics after they stop drinking [132].

### Objective measurements

Objective measurement of sleep in persons with alcohol use disorders confirms self-reported sleep problems in many respects, and provides additional insight into the nature of the underlying sleep abnormalities.

#### Sleep latency (SL)

Although it is known that alcohol can decrease sleep latency when consumed by healthy persons [124], chronic use leads to increased sleep latency, consistent with individual self-report. Published studies show that SL is prolonged during periods of drinking [9, 33, 85, 199, 221], during acute withdrawal (e.g., weeks 1 and 2 of abstinence) [9, 33, 85, 199, 221], and during post-acute withdrawal (e.g., weeks 2 through 8) [33], (Table 2) with evidence for sleep latency prolongation in inpatient and outpatient settings (e.g., [83, 123, 183]), and when controlled for age and sex, among other variables [26]. After the second month of abstinence, sleep latency may still be increased [213], or normalized [174], with evidence for normalization also present after five [69] and 9 months of abstinence [213].

**Table 2:** Objective sleep changes during early and late abstinence, in comparison to healthy sleepers

	Alcohol		Cocaine		Cannabis		Opioids	
	Early Abs	Late Abs	Early Abs	Late Abs	Early Abs	Late Abs	Early Abs	Late Abs
Sleep latency	?	↑	?	↑	↑	?	↑	↑
Total sleep time	?	↓	?	↓	↓	↓	↓	↓
Slow wave sleep	?	↓	↓	↓	↓	?	↓	↓
REM sleep	?	?	?	↓	↑	↓	↓	?
REM latency	?	?	↓	?	↓	?	↑	?

*Early Abs* Early Abstinence or acute withdrawal

*Late Abs* Late Abstinence or subacute withdrawal

*?* Insufficient data or conflicting results across studies

#### Total sleep time (TST)

Congruent with increased sleep latency, total sleep time is reduced in persons with alcohol use disorders during periods of drinking, acute withdrawal, and post-acute withdrawal [33, 85, 86, 199, 221], with very few exceptions [9]. Numerous studies examining total sleep time from 2 to 4 weeks of abstinence document reduced sleep time compared to healthy controls [69, 83, 183] (Table 2). Reduced total sleep time has also been observed in study designs that control for age and sex, among other variables [26].

Total sleep time in persons with alcohol use disorder may improve after sustained abstinence. For instance, one group found decreased yet gradually improving TST among alcoholic subjects after 19 weeks, 14, and 27 months of abstinence (312, 335, and 349 min, respectively) [69]. Another study examined TST after 1–2 years of abstinence and found no abnormalities among subjects recruited from Alcoholics Anonymous vis-à-vis controls [2].

#### Slow-wave sleep (SWS)

Considerable evidence points to deficits in slow wave sleep time (i.e. stage 3 and stage 4 sleep, or stage N3 sleep in the newer nomenclature) or slow-wave sleep activity (i.e. EEG spectral power in the slow wave frequency range) in persons with alcohol use disorders [33]. Most of this evidence comes from studies reporting results from the first few weeks of abstinence, including acute withdrawal [196], subacute withdrawal (i.e. days 8 and 12); [106] (Table 2), and beyond [26, 69, 83, 127].

Although there is evidence that SWS deficits are recovered with prolonged abstinence, current literature does not provide a definitive time frame for these improvements, yet does suggest that it may be between 3 and 14 months [33] or longer. While one study found no difference between alcohol users and controls at 25 days abstinent [183], other studies found that SWS had improved at 3 months, and normalized at 9 months of abstinence [196]. In contrast, other studies have reported persistent deficits [69] or a trend toward deficits [2] after as long as 1–2 years of abstinence, with complete recovery occurring only after 1–4 years of abstinence [199].

Intriguingly, acute alcohol use has been shown to reverse the chronic slow wave sleep deficits observed in chronic alcohol users [33, 86]. Given the widespread importance of slow-wave sleep [65] in factors including sleep continuity, learning, and memory, as well as other types of cognitive performance, the deficits associated with chronic use (and their reversal with acute use of alcohol) suggests the particular importance of slow-wave sleep in alcohol use disorders. More specifically, as the brain processes that underlie the generation of these slow waves appear to be chronically altered by chronic alcohol use, and to be temporarily restored by acute use, this chronic alteration is implicated as a potential factor in relapse.

#### Rapid eye movement (REM)

For both persons without alcohol use disorders (AUD) [118, 219] and individuals with alcohol use disorders, drinking alcohol acutely suppresses REM sleep time. For persons with AUD, REM rebound occurs several days later [33]. An early study of sleep in persons with AUD who were exposed to alcohol found that REM sleep, measured as a percentage of total sleep time (REM%) was less, relative to baseline, after 2–3 days of abstinence, but then rebounded after 5–6 days of abstinence [7]. This rebound in REM sleep has been explained as reflecting both an increased number of REM periods as well as shorter intervals between each REM cycle [196]. REM rebound has been documented after 2–3 weeks of abstinence [26, 69, 83, 127], and even after 27 months of abstinence [69].

Notwithstanding the above findings, the literature on alcohol and REM sleep has some inconsistencies (Table 2). For example, a meta-analysis examined six studies that did not consider covariates and four studies that controlled for variables such as age and sex (all participants abstinent for at least 3 weeks). Even though the analyses among all subjects showed no differences in REM measured as the percentage of total sleep (REM%), the analyses did find increased REM% in persons with AUD compared to controls when controlling for some variables [26]. Other studies have found no difference in REM% between chronic alcohol users and normal controls in the second [106] and third [83] week of abstinence. Additionally, supporting the finding of no difference in REM between chronic alcohol users and controls, a study examining REM time after 4 weeks of abstinence found no difference between subjects with AUD and normal controls [183]. Another discrepancyappears in the form of a study that found REM% among participants with AUD to be reduced after 12 weeks of abstinence in comparison with REM% after 4 weeks of abstinence, arguing against a lasting REM rebound [171].

Possibly contributing to differences in REM sleep findings are variations across studies in how control participants were recruited [183] and how well these control subjects matched the subjects with AUD in terms of measures relevant to sleep architecture such as age. An example of this is a meta-analysis exhibiting different results depending on whether authors controlled for those variables or not [26]. Another factor could be that several studies report REM%, thus, the numbers are also a reflection of not one, but two sleep architecture measurements (e.g., REM and TST) that are both undergoing dynamic changes as subjects with AUD progress in abstinence [69].

#### REM latency

Data on REM latency in persons with alcohol use disorders is more limited but also show some discrepancies. For instance, while some studies report that REM latency is decreased during the second week of abstinence [69, 106], as well as up to two years later [69], other studies do not report differences in REM latency [26, 32] (Table 2). One potential explanation for the inconsistencies in this measure could lie in the heterogeneity of subjects with AUD with regard to co-occurring conditions like depression. Supporting this idea is the finding that AUD subjects with secondary depression exhibit shorter REM latency compared to AUD subjects who do not have secondary depression [83].

#### Objective sleep quality or consolidation of sleep

Several studies examining sleep in persons with alcohol use disorders also reported data on fragmentation of sleep. Sleep fragmentation reflects awakenings or switching from a deeper to a lighter stage of sleep, and is measured by the number of switches from one stage of sleep to another, the number of awakenings, and the time spent awake after sleep onset. The measurements of sleep fragmentation provide some insight into the objective quality of sleep. Results from these studies show consistent deficiencies in objective sleep quality, with an increase in sleep stage switches compared to healthy controls from day 2 of abstinence to as far out as 1–2 years of abstinence [2, 106, 196].

Sleep stage switches, number of awakenings and time awake after sleep onset were also found to be increased on the second night of abstinence compared to the second week of abstinence, which suggests greater abnormalities in objective sleep quality occur during the withdrawal period [106]. In addition, increased sleep fragmentation was observed after 3 and 9 months of abstinence [196].

### Relationship between subjective and objective outcomes

Although there is limited published data on the relationship between subjective and objective sleep measurement in persons with alcohol use disorders, one group studied 172 individuals with alcohol use disorders of whom 104 had insomnia as determined by the Sleep Disorders Questionnaire [35]. They found that participants with baseline insomnia had longer sleep latency and lower sleep efficiency at an average of approximately 1 month abstinent than those without, suggesting a correspondence between self-report and objective measurement.

### Clinical and laboratory correlates of subjective and objective sleep outcomes

In a small study, increased sleep latency associated with chronic alcohol use was linked with lower overall melatonin levels as well as with a delay in the onset and peak of melatonin [123]. A much larger study found an association between increased sleep latency and decreased sleep efficiency among persons with AUD and sleep disorder breathing [6].

In patients with AUD, insomnia is also correlated with amount of alcohol use [22], severity of alcohol use disorder [35], and self-report of alcohol use as a sleep aid [35]. An association between insomnia and severity of self-reported depression symptoms has also been recognized [35].

#### Relationship between subjective measurements and clinical outcomes

Several studies among alcohol AUD subjects have documented the relationship between self-reported insomnia and clinical outcomes. These studies examined the effects of 1 week of abstinence while undergoing inpatient admission (e.g., subjects who self-reported insomnia during this week had higher likelihood of choosing to drink as part of a subsequent inpatient period in which this option was allowed) [182], and showed similar results after following subjects for 3 months post-detoxification (e.g., compared to subjects who did not relapse during this period, subjects who did relapse were more likely to answer “yes” to the statement, “It takes me a long time to fall asleep” from the Nottingham Health Profile [NHP] or, “I sleep badly at night,” as part of their baseline assessments) [76], or after following subjects for 5 months (e.g., having insomnia based on the Sleep Disorders Questionnaire after 2 weeks of abstinence was a predictor of relapse). Also, subjects who relapsed after 5 months had more baseline complaints of difficulties falling asleep and abnormal sleep than the group who did not relapse [34, 35].

#### Relationship between objective measurements and clinical outcomes

Mixed findings implicate objective sleep measurements as predictors of clinical outcomes in AUD. For example, increased sleep latency measured within the first 2 weeks of inpatient admission increased the odds of relapse to alcohol use within the following 5 months [34]. Similarly, increased sleep latency and decreased sleep efficiency after 16 days and 19 weeks of abstinence were associated with lower rates of abstinence at 14 months [69]. However, one study found no difference in sleep latency at 5 days abstinent between persons who subsequently relapsed and those who remained abstinent [82].

Two studies have reported a connection between slow-wave sleep and clinical measures in persons with AUD. Slow-wave sleep time was inversely correlated to the maximum number of withdrawal symptoms reported during subacute withdrawal (8–32 days of abstinence) [83], and lower percent of stage 4 NREM sleep was associated with relapse [34]. However, another study found no difference in slow-wave sleep between relapsers and abstainers [82].

Similar to clinical studies examining sleep latency and SWS, REM sleep measurements appear to be important in clinical outcomes, but with conflicting results. The differences observed here might be consistent with the differences in the measurement of REM sleep in persons with AUD described above. For instance, while one study indicated a positive correlation between low REM% with response rate in a button-press task to obtain an alcoholic drink [8], Gillin et al. showed increased REM% and shorter REM latency upon admission and upon discharge from a four-week admission among relapsers in comparison with abstainers [82]. Another study showed that increased REM latency decreased the odds of relapsing [34], and one study found no connection between REM latency measured at 19 weeks of abstinence and subsequent relapse [69]. The variation in results regarding REM sleep may be due to the different effect that acute and chronic use, have on REM sleep, and be due to changes in REM sleep as the number of days abstinent increases. Another important consideration is that achieving long periods of abstinence (e.g., like 19 weeks) is in general a good predictor of abstinence and does so to a much greater degree than the predictive qualities of other physiological measurements obtained early in abstinence.

### Pharmacotherapy options targeting sleep abnormalities

Because of the profound effects of chronic alcohol use and sleep and the apparent connection between sleep measures and clinical outcome, several studies have examined the role of sleep-promoting medications in treating persons with AUD (for review see [119]). In double-blind, placebo-controlled and other trials, gabapentin has been studied with largely [112, 136, 137] but not entirely [39] positive results. These studies suggest that gabapentin may promote both sleep outcomes and abstinence [137] in persons with alcohol use disorders.

Given the suggestion that melatonin levels are decreased in alcoholics [177, 212], the melatonin receptor agonists ramelteon and agomelatine have been examined in case series. Among patients who had been abstinent for 2–13 weeks, ramelteon was associated with decreased scores on the Insomnia Severity Index (ISI),decreased sleep latency, and increased total sleep time measured by actigraphy [36]. Similarly, agomelatine was associated with improved sleep as measured by the Pittsburgh Sleep Quality Index after 6 weeks [87].

Another potential pharmacotherapeutic agent that has been studied in this population is quetiapine. In a double-blind, placebo-controlled trial, quetiapine was associated with improvements in time awake after sleep onset and subjective insomnia [53]. In addition, a retrospective study showed an improvement on the insomnia subscale of the HAM-D [173] with quetiapine. The effect of quetiapine on alcohol-related clinical outcomes has been mixed, with evidence for improvement in abstinence rates in one study [142], and increased risk of re-hospitalization in another [143].

Trazodone is widely prescribed as a sleep aid in persons with addictions because of its lack of addictive potential. Although studies of trazodone in persons with AUD has shown benefits in sleep measurements [125] in a large, placebo-controlled trial, those benefits on sleep quality did not result in clinical improvement, but rather trazodone was associated with less abstinence during treatment and an increase in drinking after cessation of treatment [79]. Findings like these suggest that the relationship between sleep physiology and alcohol use and relapse is not simple. Rather, treatments directed at sleep that improve qualitative sleep but do not address the underlying physiological changes associated with chronic alcohol use may not be expected to promote abstinence.

Unlike trazodone, the popular benzodiazepine and benzodiazepine-like agents are often avoided in persons with alcohol use disorders because of their addictive potential and the increased risk of toxicity or overdose when these medications are mixed with alcohol [16, 90].

## Cannabis

### Subjective measurements

Like alcohol, cannabis may improve subjective sleep complaints [56], particularly when used over short periods of time. For instance; in studies using self-report questionnaires (e.g., Leeds Sleep Evaluation Questionnaire) participants report greater ease in getting to sleep [50]. However, like alcohol, chronic cannabis use is associated with negative subjective effects on sleep that are manifested most prominently during withdrawal. Notably, these subjective effects are present during discontinuation of cannabis use even among persons who were exposed to low dosages [97], and are common among regular users [61, 188]. Symptoms reported include sleep difficulties [61] such as strange dreams, insomnia, and poor sleep quality. Such symptoms occur in anywhere from 32 % [58] to 76 % [27, 222] of persons experiencing withdrawal. These studies have been conducted in both the inpatient (residential) [61, 97, 98] and outpatient levels of care [42, 44], and in studies with as many as 450 participants [222]. Placebo-controlled studies have examined what happens after discontinuation of oral THC use [97] or after discontinuation of smoked marijuana [98]. Regardless of design, studies of the effects of chronic use have consistently shown reliable and significant changes in subjective reports of sleep during abstinence in comparison to baseline [42].

Among the problems with sleep in chronic cannabis users is the presence of strange dreams [44]. Such dreams typically begin 1–3 days after cannabis discontinuation—when sleep quality is particularly poor [42, 44, 195], peak after 2–6 days, and last 4–14 days [44], coincident with other subjective sleep complaints. However, large studies have found sleep difficulties lasting for longer periods, such 43 days [58], and strange dreams in particular lasting for as long as 45 days [44]. Returning to cannabis use (or using alcohol or other sedatives) to promote sleep is commonly observed [58].

However, the sleep-promoting effect of cannabis is lessened in the chronic user compared to naïve users [50–52, 91], while the negative effects of cannabis on sleep intensify with chronic use as noted above. This scenario leaves the chronic user in a potential catch-22: heavier use of cannabis may be necessary to receive its subjective sleep-promoting effects in the chronic user, but at the same time this increased use contributes to worsening overall sleep and therefore leads to continued and greater use.

### Objective measurements

Studies examining the effect of cannabis on objective sleep measurements obtained either by an experienced observer rating sleep by polysomnography (PSG) largely confirm the subjective reports. For instance, an observer-rated study showed that administration of 10, 20, or 30 mg of THC decreased total time to fall asleep [60], and a PSG study showed both shorter sleep latency (SL) [150], and decreased time awake after sleep onset (WASO) [160]. However, other studies have not observed a decrease in sleep latency or wake time after sleep onset [75]. One possible explanation for the difference in findings may be related to disparate effects of THC (sleep promoting) and cannabidiol (a non-euphorigenic cannabinoid preferred in some medical preparations), which may increase alertness [150].

Several studies of PSG-measured sleep report increased SWS [25, 75], decreased REM sleep [74, 75, 160], and decreased REM density (e.g., number of eye movements during REM sleep) [74, 75]. However, this pattern is not always replicated [150].

In chronic users of cannabis, the effects of cannabis on objectively measured sleep are notably different. With chronic use, individuals develop tolerance to most of the effects observed in naïve users, including its sleep-inducing effects and slow-wave sleep enhancement [25, 78, 111, 163]. Sleep efficiency is similarly unimproved [163] or worsens [111]. The tolerance to REM sleep changes, however, appears to be relatively muted [75]. However, no consensus exists with respect to REM time and studies have reported decreased, no change [163], or increased [111] REM time.

PSG studies of cannabis withdrawal have demonstrated increases in sleep onset latency and wakefulness after sleep onset [27, 28, 75, 77, 78, 175] (Table 2). Total sleep time, sleep efficiency, and slow-wave sleep time is reduced [1, 27, 28, 75, 78] (Table 1), and REM sleep is increased (REM rebound) [74, 75, 77, 108, 160, 175]. Shorter REM latency has also been reported [27, 77].

Changes in the objective PSG measurements during withdrawal can start as soon as the first night of abstinence (e.g., the decrease in SWS time [74]). Changes during withdrawal are more noticeable among heavy marijuana users (marijuana use ≥5 times per week over the past 3 months) [27]. With continued abstinence, TST, SE, and amount of REM sleep decline (Table 2), while WASO increases. These disturbances progress over the first 2 weeks of abstinence [28, 44, 120] and persist for more than 45 days into a marijuana abstinence period [44].

There are conflicting reports with regard to REM sleep in sustained abstinence. Initially it appears that REM sleep time increases/rebounds early in abstinence, but decreases as abstinence progresses [28] (Table 2). The reason for this continued worsening of sleep with decreasing REM sleep during abstinence is unclear, but could reflect a pre-existing, underlying sleep problem and/or the long-term effects of chronic use.

### Relationship between subjective and objective outcomes

As noted above, the desirable effects of cannabis on sleep are reported less frequently in chronic cannabis users compared to naïve users [91], Chronic users also report difficulty sleeping and strange dreams among other symptoms associated with abstinence [11, 28, 43, 58, 175, 194]. These subjective findings have been correlated to longer sleep onset latency, reduced slow-wave sleep, and REM rebound observed in PSG studies [27, 198].

### Clinical and laboratory correlates of subjective and objective sleep outcomes

Sleep difficulties appear to be a predisposing factor for cannabis use, and baseline sleep problems are a significant predictor of later cannabis use, doubling the risk of future use [139, 155, 166, 211, 214]. This latter finding has led some to describe cannabis use as “coping oriented use” [21].

The sleep disturbances encountered in marijuana withdrawal may play a crucial role in treatment outcomes. Higher rates of relapse have been correlated with sleep problems and other withdrawal symptoms [43]. In a study focused on military veterans, Babson et al. showed that poor sleep quality prior to the quit attempt was a predictor of higher rates of later cannabis use [19–21]. Similarly, poor sleep quality during abstinence also contributes to relapse [44, 46, 195]. Evidence from a limited number of studies suggests that objective findings, like increased periodic limb movements during abstinence, are correlated with quantity and duration of cannabis use [28].

### Pharmacotherapy options targeting sleep abnormalities

Sleep disturbances associated with withdrawal improve with oral administration of THC or resumption of cannabis use [42, 44, 45, 95, 97, 108]. THC exerts a dose-dependent effect in reducing withdrawal symptoms [45], but as noted above, the beneficial effects of THC on sleep diminish with chronic use, and chronic use leads to more severe problems with sleep.

Haney et al. found the greatest benefit regarding sleep symptoms and relapse using combination therapy with lofexidine (an alpha-2 agonist) and oral THC. However they did not report any benefit from 10 days of oral THC alone [94]. Nabilone, a FDA-approved synthetic analog of THC, has the potential to reverse withdrawal-related irritability and disruptions in sleep, and promotes abstinence [92]. Nabiximols, a synthetic combination of THC and cannabidiol has a non-significant positive effect on these parameters [10].

The use of valproic acid resulted in no benefit and even some worsening of symptoms in chronic cannabis users [95, 128]. No definitive benefits have been reported with Lithium [107], nefazodone [96], or bupropion [49, 99].

Subjective and/or objective sleep parameters have been shown to improve with the use of zolpidem [195], mirtazapine [93], gabapentin [135], and quetiapine [57], but none of these agents have conclusively reduced the relapse rate.

## Cocaine

### Subjective measurements

Withdrawal from cocaine is characterized by numerous subjective complaints, including sleep and sleep-related complaints. The first several days to 1 week after cocaine cessation are characterized by sleep disturbances, hypersomnia, bad dreams, depressed mood, psychomotor agitation and retardation, fatigue, and increased appetite [38, 59, 80]. With continued abstinence, however, there is subjective improvement of sleep as well as improvements in other cocaine withdrawal measures [209], with apparent normalization of subjective sleep over the course of several weeks [80].

Numerous studies have indicated an improvement in self-reported sleep quality over the first few weeks of abstinence [13, 55, 81, 138, 147, 148, 153, 172, 209], with improvements in measures such as overall sleep quality, daytime alertness, concentration/confusion, depth of sleep, and energy/fatigue. However, the possibility that such improvements may be related to acclimation to a new environment (e.g., the treatment setting [209]) and not actually reflect good sleep relative to healthy persons [55] has been raised. Possibly providing some answers to these questions, a laboratory study that included self-administration of cocaine either early or late in a 3-week period of abstinence showed that subjective sleep quality was at its worst in the first few days following cocaine use and improved with continued abstinence [148]. In addition, whereas chronic cocaine users show impairment in self-reported but quasi-objective sleep measurement like the Pittsburgh Sleep Quality Index (PSQI), visual analog scale ratings of subjective sleep quality in the third week of abstinence are no different from healthy sleepers [147]. Hence there is evidence that chronic cocaine users have chronically impaired sleep as measured by instruments like the PSQI, that self-reported sleep quality improves with continued abstinence, and that self-reported sleep quality after an extended period of abstinence is similar to that in healthy sleepers. However, this last finding may only show that chronic cocaine users’ intrinsic scale for self-report of sleep quality is different from healthy sleepers, with ‘good’ subjective sleep in chronic cocaine users seemingly good only in comparison with the much worse sleep experience they have at other times.

### Objective measurements

Although self-reported sleep improves following the initial withdrawal from cocaine, polysomnographic findings have consistently shown deterioration in sleep to insomnia-like levels in the same period [13, 81, 104, 121, 138, 145, 148, 152, 192]. The co-occurring deterioration in PSG-measured sleep and improvement in self-reported sleep quality was termed ‘occult insomnia,’ as poor sleep as measured by PSG was associated with poor performance on sleep-dependent learning and other cognitive tasks [148, 149]. These findings suggest that the PSG-measured deterioration in sleep and not the subjective improvement in sleep better reflects what is happening during abstinence from chronic cocaine use, and supports the notion that the intrinsic, subjective scale used by chronic cocaine users to report sleep quality is altered relative to healthy persons.

#### Sleep latency

Acute cocaine administration can increase sleep latency [104, 162, 207], but the first few days of abstinence from cocaine in chronic users is associated with short sleep latencies relative to later in abstinence [81, 121, 138, 148, 153, 192], when sleep latency may be as long as 30–60 min or more (Table 2).

#### Total sleep time

Total sleep time during abstinence is reduced in chronic cocaine users [147] but appears to be at its greatest sometime in the early abstinence period (first week of abstinence) in laboratory studies including cocaine self-administration [148]. Total sleep time decreases with continued abstinence (Table 2), however [81, 104, 121, 138, 147–149, 153, 162, 192, 207], with total sleep times around the third week of abstinence as low as 300–330 min despite prohibitions against daytime napping and the opportunity to sleep 8 h or more. Sleep efficiency follows a similar pattern, with insomnia-like levels apparent in the third week of abstinence [153]. Limited evidence suggests that chronic cocaine users able to maintain outpatient abstinence for as long as 54 days show some improvement in total sleep time [13].

#### Slow-wave sleep

Chronic cocaine users appear to have dramatically diminished slow-wave sleep time relative to age-matched healthy sleepers [13, 147] (Table 2). More limited evidence suggests that slow-wave activity is increased by cocaine self-administration earlier in the day, with a subsequent loss of slow-wave activity in the first several days of abstinence followed by a rebound over the next 2 weeks of abstinence [148]. More substantial evidence indicates that slow-wave sleep time increases modestly from the first to the third week of abstinence [138], but at 3 weeks of abstinence is still 50 % less than age-matched healthy sleepers [13]. This deficit in slow-wave sleep generation is associated with impaired slow-wave sleep specific, sleep-dependent learning [149], and is consistent with or more profound than similar findings in chronic users of alcohol, cannabis, other stimulants, and heroin [25, 27, 175, 192] suggesting an abnormality in sleep homeostasis [145] that may be common to chronic, regular use of addictive substances.

#### Rem

Cocaine administration acutely suppresses REM sleep [104, 162, 207], with a subsequent rebound evident as an increase in REM sleep time and/or percent of total sleep time spent in REM (REM%), and a decrease in REM latency [81, 104, 121, 149, 153, 192, 207]. However, in chronic cocaine users, REM sleep decreases following the rebound, with low REM times observed during the second and third weeks of abstinence [13, 104, 138, 147, 149, 153, 192] (Table 2). This diminished REM sleep time is associated with cognitive consequences like poor procedural learning [149], suggesting an abnormality of REM homeostasis during abstinence from chronic use. Consistent with this idea is the observation that REM latency is higher in the third week of abstinence relative to the first [149] and at 3 weeks abstinence does not differ substantially from healthy sleepers [147] (Table 2), despite low REM sleep time.

### Relationship between subjective and objective outcomes

What is now clearly shown to be a mismatch in subjective and objective experience during acute and subacute abstinence was once perceived as an inconsistency [104, 153]. One possible cause for the mismatch may be dysregulation of the homeostatic sleep drive in chronic cocaine users, wherein the ‘sleepiness’ and other negative effects of increased wakefulness are not experienced subjectively [148]. Additionally, or alternatively, the rebound in delta power after acute withdrawal [148], despite poor sleep and decreased slow-wave sleep time, may improve the subjective experience of sleep quality [122, 148]. The poor subjective experience in acute withdrawal may also be related to the decreased REM latency and increased REM sleep time, leading to increased dreaming [38, 59] and correlated with symptoms of withdrawal [13].

### Clinical and laboratory correlates of subjective and objective sleep outcomes

#### Cognitive correlates of sleep outcomes

Chronic cocaine use is associated with various cognitive performance deficits (e.g., see [152]) that may predict treatment retention and other outcomes [3–5]. As described briefly above, poor sleep associated with abstinence from chronic use may contribute to poor cognitive performance including decreased attention or vigilance [148, 149, 152]. The most direct associations between poor sleep and cognitive deficits, however, are observed in sleep-dependent procedural learning. In such tasks, overnight learning is strongly correlated with objective sleep measurement, such as slow-wave sleep time [189], REM time [189], and stage 2 (N2) sleep time (M. P. [202]. In chronic cocaine users, similar correlations are present; in nights with relatively normal sleep, normal sleep-dependent learning takes place, but in nights with impaired sleep, such learning is similarly impaired [148, 149]. Hence, sleep abnormalities associated with abstinence from chronic cocaine use may be responsible for significant impairment in normal, sleep-dependent learning, as well as more immediate cognitive function like attention. Intriguingly, cocaine administration is associated with temporary reversal of these deficits [148, 149, 152], further implicating such deficits in risk for relapse.

#### Relationship between objective measurements and clinical outcomes

Recent evidence supports the assertion that poor sleep associated with abstinence from cocaine not only impairs cognitive performance, but also contributes to increased cocaine use or relapse [13]. In this study, the homeostatic response to continued abstinence (which was measured as change in slow-wave sleep time from the first week of abstinence to the second or third week) predicted the amount of cocaine self-administered in a laboratory experiment and clinical outcome in a clinical trial. In addition, REM sleep time and total sleep time during the third week of abstinence predicted the amount of cocaine self-administered. In all cases, improvements in sleep were associated with less self-administration or better clinical outcome.

### Pharmacotherapy options targeting sleep abnormalities

Although several medications such as modafinil, topiramate, tiagabine, gaboxadol and vigabatrin [145] have been suggested as potential options for targeting the sleep abnormalities associated with chronic cocaine use, few studies have examined the effects of medications on sleep in chronic cocaine users, and several of the suggested medications have potentially significant safety issues (i.e. tiagabine, gaboxadol, and vigabatrin). Hypothesizing that the REM rebound associated with initial withdrawal from cocaine was caused by dopamine insufficiency [62], Gillin et al. [81, 82] examined the effect of lisuride, a high affinity dopamine D2,3,4 receptor agonist, on sleep. While lisuride had the desired effect on REM sleep (decreasing REM% and increasing REM latency), it had no effects on other withdrawal-related phenomenon [81]. In light of the clinical findings in Angarita et al. [13] and the effect of prolonged abstinence on REM sleep, it seems unlikely that reduction in REM sleep time would be beneficial during extended abstinence, but *increasing* REM to normal values could be beneficial.

Another medication that has been tested is tiagabine, a GABA-reuptake inhibitor. Since tiagabine is known to increase slow-wave sleep time, which is implicated in improved cognitive performance in sleep-restricted persons [203], it was hypothesized that tiagabine may improve slow-wave sleep time in chronic cocaine users [146]. While tiagabine had dramatic effects on slow-wave sleep time, sleep architecture appeared unnatural, with slow-wave sleep occurring throughout the sleep period. Additionally, there was no apparent benefit to total sleep time, and no consistent benefit in cognitive performance [146].

Perhaps the most promising, and most studied medication to be tested for correcting sleep abnormalities related to cocaine is modafinil. A stimulant and cognitive enhancer that appears to act at least partially through dopamine transporter blockade, modafinil appears to share some important properties with cocaine while being a relatively safe medication with low abuse potential [140]. In chronic cocaine users, modafinil has been shown to normalize slow-wave sleep time, as well as other sleep parameters [147]. Though effects of modafinil on clinical outcome have been mixed (e.g., [12, 63, 64, 176]), its effects on sleep and its pro-cognitive effects position it as the best candidate at present for a viable pharmacotherapy for cocaine use disorders.

## Opioids

### Subjective measurements

Short-term opioid use can cause sedation and daytime drowsiness [130, 159, 216, 217]. Dizziness and sleepiness are common side effects of opioid pain medications [41, 109]. With a stable dose, tolerance to the subjective, sedative effects of opioids develops within 2–3 days and some studies find that cognition normalizes after that [103, 129], supporting the notion of tolerance to the sedative effects. However, there is also evidence that unpleasant sedative effects, decreased alertness and increased reaction time in a variety of cognitive tasks continue to be experienced by some patients on a stable dose of narcotic medication [23, 24, 54, 181]. These differences in findings may be related to inconsistencies in how the sedative effects are defined [217].

Xiao et al. [215] studied the quality of sleep in persons with heroin use disorder on early methadone maintenance therapy (MMT) after a median of 5.4 days of treatment [215]. Patients without pre-existing chronic sleep disturbances demonstrated lower ratings of sleep (Pittsburgh Sleep Quality Index [PSQI]) and daytime sleepiness (Epworth Sleepiness Scale [ESS]) compared to healthy sleepers. Oyefeso et al. [151] reported inadequate sleep quality and quantity as well as difficulty initiating and maintaining sleep in persons with opioid use disorders in early stages of methadone detoxification. Similar studies have shown some increased daytime drowsiness and below normal sleep measures in this patient population [113, 114, 134, 204]. After longer periods of MMT, however, there is some degree of tolerance to these effects [206], and sleep difficulty is shown to be present only in the first 6–12 months of MMT [158, 193].

There is a limited number of reports studying the effects of withdrawal and abstinence from chronic opiate use. Asaad et al. reported insomnia, hypersomnolence, increased sleep latency, and reduced sleep duration in individuals with opioid use disorder after 3 weeks of abstinence [17].

### Objective measurements

Sleep architecture in healthy adults can be significantly altered even after a single dose of oral opioids [67]. Using electroencephalography (EEG) and electromyography (EMG), Kay et al. [117] reported that acute intoxication with heroin, morphine, or methadone resulted in dose-dependent enhancements in arousal during sleep–wake periods. Heroin use demonstrated a stronger effect particularly on reduction of theta waves and REM sleep [117, 204]. Morphine and methadone reduce slow-wave sleep and in-crease stage 2 sleep [67]. Several studies have shown that acute use of various opioids results in increased REM latency [115, 159], decreased REM sleep time [113–117, 130, 159, 180], increased stage 1 [67, 130, 180] and stage 2 sleep [67], and decreased slow-wave sleep [113–117, 159]. Acute use of opioids also leads to increased sleep latency [116, 130], increased wakefulness after sleep onset (WASO) [113–117, 130, 159, 215], and concomitant decreases in total sleep time (TST) [116, 215] and sleep efficiency (SE) [116, 159, 215].

There is a partial tolerance to the effects of opioids with some evidence for increased REM sleep time in acute use [113, 114, 130], and less pronounced changes in SWS, wakefulness, and arousal observed after chronic use. However, vocalization during REM sleep, delta bursts, and increased daytime sleepiness may be observed in this phase [204]. Tolerance to sleep problems is more prominent in MMT [113, 134, 159, 204], with evidence that persons in treatment for more than 12 months exhibit better recovery sleep following sleep deprivation than persons in shorter-term treatment [193].

Nevertheless, abnormal PSG findings are commonly reported in chronic opioid users despite development of tolerance. These abnormalities include increased sleep latency [100, 185], increased awakening [100, 113, 185, 190], decreased total sleep time [100, 185], and decreased sleep efficiency [100, 190]. Slow-wave sleep time [113, 114, 185, 190] and REM sleep are decreased compared to baseline [113, 185, 190, 205], while duration of stage 2 sleep is increased similar to acute use [190, 205]. Analysis of actigraphy data from patients with prescription opioid use disorders indicated poor sleep in terms of total sleep time, sleep efficiency, sleep latency, total time awake, and time spent moving [100].

Several studies have reported changes in patterns of sleep with progressive abstinence from opiates. At around 5–7 days of acute abstinence from chronic heroin use, Howe et al. reported decreased total sleep time, slow-wave sleep, REM, and stage 2 sleep and increased sleep latency, wake after sleep onset, and REM latency compared to healthy sleepers [101] (Table 2). During the first 3 weeks of abstinence, prolonged sleep latency, decreased sleep efficiency, decreased TST, increased arousal index, increased stage 1 and 2, and decreased slow-wave sleep (SWS) were prominent compared to healthy sleepers [17] (Table 2). After 6 weeks and up to 6 months of abstinence from methadone, there is a rebound increase in SWS and REM time to a higher level than baseline [113, 114, 134].

### Relationship between subjective and objective outcomes

Using PSG data, Xiao et al. showed an inverse relationship between the Epworth Sleepiness Scale (ESS) scores and SWS time in patients with heroin use disorder who were in early methadone treatment [215]. They reported poor initial quality of sleep based on the PSQI scores which were significantly correlated with their methadone dosages [102]. PSQI score were also found to be significantly correlated with average diary-reported sleep time, subjective ratings of feeling rested, and PSG sleep efficiency in MMT patients [179]. Overall the high prevalence of sleep complaints in this population along with documented abnormal objective findings argue that these complaints are more likely to be secondary to pathology rather than sleep misperception.

### Clinical and laboratory correlates of subjective and objective sleep outcomes

#### Opioids and sleep disordered breathing

Acute use of small doses of opioids does not appear to significantly increase the risk for increased sleep-disordered breathing [167, 180]. However, chronic opioid use has been associated with several abnormalities including nocturnal oxygen desaturations, abnormal breathing patterns, and Biot’s respiration pattern which ultimately may lead to hypercapnia and hypoxia [29, 73, 88, 126, 164, 165, 191, 201, 204, 218]. Chronic opioid treatment, particularly with extended release preparations is associated with increased risk of central and obstructive sleep apneas compared to BMI and age-matched controls [73, 88, 141, 190, 200, 201, 204, 205, 208]. Between 30 and 90 % of patients on chronic opioid therapy display signs of central apnea in a dose-dependent fashion [73, 190, 200, 201, 205]. Several studies have indicated that chronic opioid use is an independent risk factor for irregular breathing, central apneas, and hypopneas [88, 154, 200, 201]. Additional abnormalities associated with MMT include sleep-disordered breathing, lower arterial oxygen saturation and higher carbon dioxide concentration [178, 190, 205]. There is a positive correlation between the duration of MMT and plasma methadone levels with frequency of sleep apnea [190, 205].

A multivariate analysis of the relationship between demographic factors, mental health and drug use with sleep disturbances on 225 MMT patients found that depressive and anxious symptoms, greater nicotine use, bodily pain, and unemployment were all significant predictors of poorer global sleep quality [186].

Using PSG, Asaad et al. found that severity of depression in MMT patients was inversely correlated with SWS. They reported that SWS in moderate and severe depression was significantly lower than in milder depressive states. However, duration of opioid abuse or type of opioid did not show a significant correlation with the abnormalities in the sleep profile [17].

#### Relationship between subjective measurements and clinical outcomes

Peles et al. [156] used a logistic regression model and showed that a higher methadone dose (defined as greater than 120 mg/day) was associated with poor sleep quality, higher rate of sleep disturbance, more frequent use of sleeping medications, and higher rate of daytime dysfunction [156]. However in a later study (2009), they found no direct correlation between the methadone dose and worse objective and perceived sleep parameters. Rather they suggested that duration and intensity of opioid abuse before admission to MMT was directly correlated with sleep abnormalities [157].

Quality of sleep in substance users who are trying to quit plays an important role in predicting the treatment outcome and poor sleep quality is associated with higher risk of relapse [40, 204]. Predictive factors for abstinence 1 month after detoxification with naltrexone may include sleeping problems upon discharge and any changes in sleeping problems [66]. In MMT patients, psychiatric disorders, greater nicotine and benzodiazepine use, bodily pain, and unemployment are associated with poorer global sleep quality [156, 186].

#### Relationship between objective measurements and clinical outcomes

Using PSG, Peles et al. evaluated patients with heroin use disorder who were being treated with high and low dose methadone [157]. Of the objective sleep indices, percentage of non-REM deep sleep (i.e. SWS) inversely correlated with number of years of opioid abuse. They found that a lower percentage of SWS and more years of opioid abuse were observed in the group who received higher methadone dose during MMT.

#### Positive effects of opioids on sleep

Judicious use of opioid medications might improve pain-related sleep disorders [14, 30]. Subjective reports of improved sleep after pain control with extended-release morphine sulfate use to treat patients with chronic hip or knee arthritis are backed by objective evidence obtained from PSG indicating better sleep quality [170]. Opioids have also been used to treat a sleep disorder known as periodic limb movement (PLMS), which is often associated with restless legs syndrome [144].

### Pharmacotherapy options targeting sleep abnormalities

Modifiable psychological and medical risk factors associated with sleep disturbance should be identified and corrected in order to improve quality of life in drug treatment. Treatment of sleep disorders among MMT patients, particularly in those with psychiatric disorders, benzodiazepine abuse, chronic pain, and patients who are on high methadone dose is of crucial importance.

#### Methadone

Methadone maintenance is widely used and a standard pharmacotherapy for treating patients with opioid use disorders [18, 68, 71, 197, 220]. Chronic methadone use is more commonly associated with tolerance to the sleep problems compared to other opioids [113, 134, 159, 204]. However, more than three-quarters of persons receiving methadone maintenance therapy (MMT) still report sleep complaints [151, 156, 186]. This is complicated by the fact that about 50 % of MMT patients report use of both illicit drugs and legal medications to help with sleep [156, 186]. Methadone and electrostimulation (ES) have been used to treat insomnia in the first 30 days of opioid withdrawal [84]. In the first 2 weeks of withdrawal patients treated with electrostimulation had shorter sleep time and more awakenings than patients receiving methadone. They also found that subjects in the ES group who remained in treatment experienced more sleep disturbance than those who dropped out prematurely. Overall methadone and ES were not efficient in treating insomnia associated with withdrawal. Stein et al. tested whether trazodone (50 mg/night), one of the most commonly prescribed medications for treatment of insomnia, improved sleep among methadone-maintained persons with PSQI score of six or higher [187]. They found that trazodone did not improve subjective or objective sleep problems in this group of patients.

#### Buprenorphine

Buprenorphine was FDA approved as a pharmacotherapy for opioid use disorders in 2002. Buprenorphine has the advantage of being available from office-based practices [131]. There are limited numbers of studies looking at the effect of buprenorphine on sleep. One study suggests that buprenorphine is comparable to methadone in improving sleep quality in patients involved in long-term treatment [133]. In another study, forty-two patients with opiate use disorder were treated with either methadone or buprenorphine and gradually tapered down over the course of 2–3 weeks. Buprenorphine-treated patients had 2.5 % lower sleep efficiency and 9 % shorter actual sleep time. These significant group differences were most pronounced with the lowest doses toward the late withdrawal phase [161]. The time course of tapering buprenorphine during detoxification might also play a role in the quantity of sleep. A randomized controlled trial of buprenorphine for detoxification from prescription opioid use evaluated sleep time among patients assigned to receive 1, 2, and 4-week buprenorphine tapers. The 4-week taper group reported significantly less loss of sleep compared to the other groups [70].

In a study of 70 patients with chronic opioid use, the effect of buprenorphine on sleep disordered breathing was measured polysomnographically [72]. Patients in this study tended to be young (mean age of 31.8) and non-obese (mean body mass index 24.9 ± 5.9). However, treatment with buprenorphine was associated with mild to severe sleep-disordered breathing in this population, with a substantial rate of associated hypoxemia [72].

Although information on the effect of other medications on sleep in chronic opiate users is limited, Srisurapanont and Jarusuraisin [184] explored the effect of amitriptyline (57.7 ± 12/night) versus lorazepam (2.1 ± 0.5/night) to treat insomnia in 27 patients with opioid withdrawal in a randomized double-blind study [184]. The Sleep Evaluation Questionnaire and three insomnia items of the Hamilton Depression Rating Scale were used to assess sleep. All aspects of sleep (including ease of getting to sleep, perceived quality of sleep, integrity of early morning behavior following wakefulness and Hamilton Depression Rating Scale insomnia items), except for ease of awakening from sleep, were not significantly different in the two treatment groups. These findings suggest that apart from the hangover effect, amitriptyline is as effective as lorazepam in the treatment of opioid-withdrawal insomnia.

## Conclusion

Overwhelming evidence points to chronic alterations in sleep from chronic use of addictive substances that may be distinct from some or all of the acute effects of those substances. Interestingly, the effects of chronic use on sleep are similar among both CNS stimulants and depressants. Decreased sleep time, increased sleep latency and wake time after sleep onset, and deficiency in slow-wave sleep generation appear to be common to chronic use of alcohol, cocaine, cannabis, and opiates. REM sleep is also affected by acute and chronic use, but may be more sensitive to the pattern or quantity of recent use and time from last use, as results vary more among studies. Also linking these abnormalities are connections with ongoing use and relapse. However, treatment with typical sleep promoting agents that increase sleep time or efficiency by increasing light sleep may be counterproductive. Agents that address deficiency in slow-wave sleep generation and alterations in REM sleep may prove to be more useful in addressing the connection between chronically-altered sleep physiology and ongoing use and relapse, but substantial research still needs to be done to explore this possibility.
